# Supplementary material for: Thermodynamic signatures of diagonal nematicity in RbFe2As2 superconductor
Source: PNAS Nexus. 2025 Apr 4;4(4):pgaf060. doi: 10.1093/pnasnexus/pgaf060 (PMC11969064; doi:10.1093/pnasnexus/pgaf060)
Supplement: pgaf060_Supplementary_Data [file pgaf060_supplementary_data.pdf]

Supplementary Material: Thermodynamic Signatures of Diagonal Nematicity in $RbFe_2As_2$ Superconductor

Y. Mizukami[1,2,*] O. Tanaka[1], K. Ishida[1,†] A. Onishi[1], Y. Kageyama[1], M. Tsujii[1], R. Ohno[2], N. Kimura[2], T. Mitsui[3], S. Kitao[4], M. Kurokuzu[4], M. Seto[3,4], S. Ishida[5], A. Iyo[5], H. Eisaki[5], K. Hashimoto[1], and T. Shibauchi[1]

[1]*Department of Advanced Materials Science, University of Tokyo, Kashiwa, Chiba 277-8561, Japan*

[2]*Department of Physics, Tohoku University, Sendai, 980-8578, Japan*

[3]*National Institutes for Quantum Science and Technology, Sayo, Hyogo 679-5148, Japan*

[4]*Institute for Integrated Radiation and Nuclear Science, Kyoto University, Kumatori, Osaka 590-0494, Japan*

[5]*Research Institute for Advanced Electronics and Photonics, National Institute of Advanced Industrial Science and Technology, Tsukuba, Ibaraki 305-8568, Japan*

(Dated: February 20, 2025)

* mizukami@tohoku.ac.jp

† Present address: Institute for Materials Research, Tohoku University, Sendai, 980-8577, Japan

I. MATERIALS AND METHODS

A. Single crystal growth

Single crystals of $RbFe_2As_2$ were grown by the FeAs self-flux method. FeAs and RbAs were prepared as precursors by the method described in Ref. [1], weighed at a ratio of 4 : 1, and put into an alumina crucible, then sealed in a tantalum container using an arc-welding chamber. The Ta container was sealed in an evacuated quartz tube to protect Ta from oxidation. The container was heated up to 1100°C, kept for 5 hours, and then slowly cooled to 950°C for 50 hours.

B. Heat capacity

The heat capacity of the $RbFe_2As_2$ crystal is measured using the long-relaxation method, whose experimental setup is introduced in Ref. [2]. A single bare chip of Cernox resistor is used as the thermometer, heater, and sample stage, which is suspended from the cold stage by silver-coated glass fibers so that the bare chip has a weak thermal link to the cold stage as well as an electrical connection for the sensor reading. The temperature range measurable in this system is from 0.5 K to $\sim$ 60 K due to the sensitivity of the resistor. The mass of the sample used for field-angle resolved heat capacity measurement is 10.5 μg. The lateral size of the sample is $\approx 300 \times 300$ μm^2, and the thickness is 20 μm. The sample is mounted on the bare chip using Apiezon N grease. The heat capacity of the crystal is obtained by subtracting the heat capacity of bare chip and grease from the raw data. The measurement cell is placed in the center of the vector magnet, whose horizontal and vertical magnetic field components are controlled independently. The Fe-As direction of the crystal, which is determined by X-ray diffraction, is aligned manually near the $\phi = 0°$ direction of the setup. This setup inevitably gives a small offset angle ϕ_0 between $\phi = 0°$ and the Fe-As direction.

C. ^{57}Fe Mössbaur spectroscopy

The ^{57}Fe Mössbaur spectroscopy is performed using synchrotron Mössbaur source at BL11XU of SPring-8, where the details of the experimental method is described in Ref. [3]. A single crystal with the lateral size $\approx 1.3 \times 0.9$ mm^2 is used for the measurement. The

sample is weakly sandwiched by thin polyimide films which is attached to the copper-made cold stage. The sample and the stage are cooled by a He-flow cryostat.

II. ELASTORESISTIVITY TENSORS

In-plane elastoresistivity tensor components for a tetragonal crystal can be described as

$$m_{11} - m_{12} = \frac{1}{1+\nu_{[100]}} \frac{(\Delta R/R)_{[100]} - (\Delta R/R)_{[010]}}{\epsilon_{[100]}} \tag{S1}$$

$$2m_{66} = \frac{1}{1+\nu_{[110]}} \frac{(\Delta R/R)_{[110]} - (\Delta R/R)_{[\bar{1}10]}}{\epsilon_{[110]}} \tag{S2}$$

$$m_{A_{1g}} = \frac{1}{1-\nu_{[100]}} \frac{(\Delta R/R)_{[100]} + (\Delta R/R)_{[010]})}{\epsilon_{[100]}} \tag{S3}$$

$$= \frac{1}{1-\nu_{[110]}} \frac{(\Delta R/R)_{[110]} + (\Delta R/R)_{[\bar{1}10]}}{\epsilon_{[110]}}, \tag{S4}$$

which reflect the nematic susceptibility along Fe-As direction (B_{1g}), Fe-Fe direction (B_{2g}), and symmetric (non-nematic) response (A_{1g}), respectively. The $\nu_{[100]}$ ($\nu_{[110]}$) is the Poisson ratio of the piezoelectric stack, which is obtained from T-dependent strain ratio of $\epsilon_{[100]}$ and $\epsilon_{[010]}$ ($\epsilon_{[110]}$ and $\epsilon_{[\bar{1}10]}$).

Two methods have been developed to measure elastoresistivity by applying tunable uniaxial stress using piezoelectric actuators and applied for $RbFe_2As_2$; (1) two ends of the free bar-shaped sample are clamped [4, 5], and (2) the sample is affixed directly on a piezoelectric stack [6]. Method (1) is useful for the application of large strain but may have some experimental difficulties in using for mechanically delicate single crystals such as FeSe, in which one found it impossible to strain the sample homogeneously in method (1) without utilizing a specially designed substrate [7]. We find that single crystals of $RbFe_2As_2$ are also mechanically delicate and thus we choose to measure the elastoresistivity by using method (2), where one entire side of the sample is glued on the top of the piezoelectric stack. This allows us to apply uniaxial strain in the desired direction of the sample even if the crystal is mechanically soft. In our previous study [6], we have employed the longitudinal elastoresistance method with the conventional four-probe configuration and have estimated the elastoresistivity tensor only from the resistance along one direction ($R_{[100]}$ or $R_{[110]}$) for $RbFe_2As_2$ [6], which requires a condition that the A_{1g} component is negligibly small. This condition has been confirmed for $Ba_{1-x}Rb_xFe_2As_2$ with $x = 0.65$, by using the Montgomery method with four

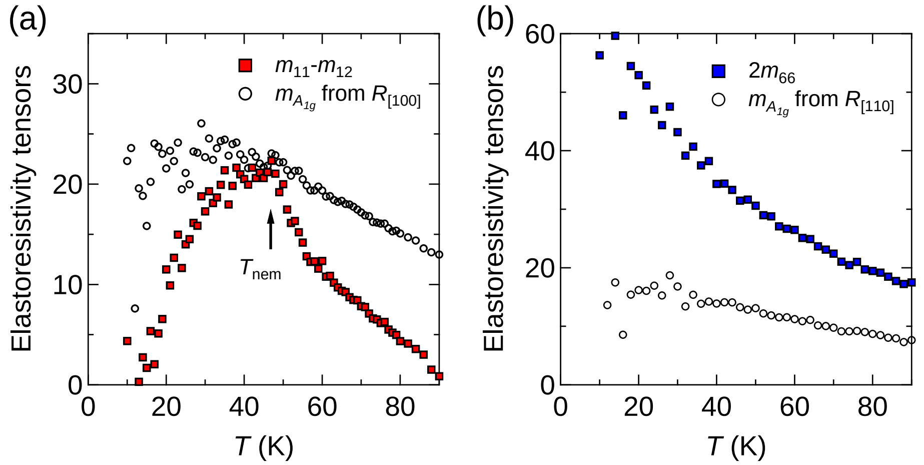

FIG. S1. Temperature dependence of elastoresistivity tensors measured by the Montgomery method in $RbFe_2As_2$. (a) Temperature dependence of $m_{11} - m_{12}$ and $m_{A_{1g}}$ measured using a crystal with anisotropic stress along Fe-As directions [100]. (b) Temperature dependence of $2m_{66}$ and $m_{A_{1g}}$ measured with anisotropic stress along Fe-Fe directions [110] using a different crystal from (a) in the same run.

contacts at the edges of the square-shaped crystals. However, recent studies of $A\mathrm{Fe}_2\mathrm{As}_2$ using method (1) report large A_{1g} component compared from other components. This calls for the revisit of the elastoresistivity tensor measurements comparing two resistance data parallel and perpendicular to the anisotropic stress direction in method (2).

Here we use the Montgomery method, from which the elastoresistivity tensor is obtained from the resistance data along two directions in a given crystal. A square-shaped sample is prepared and the resistance data along [100] and [010] directions ($R_{[100]}$ and $R_{[010]}$) are taken simultaneously to extract $m_{11} - m_{12}$ and $m_{A_{1g}}$. Similar measurements are performed to obtain $2m_{66}$ from $R_{[110]}$ and $R_{[\bar{1}10]}$ using a different crystal. These measurements for $m_{11} - m_{12}$ and $2m_{66}$ are performed in a single run by mounting two samples on a piezoelectric stack. The obtained elastoresistivity tensors $m_{11} - m_{12}$, $2m_{66}$, and $m_{A_{1g}}$ for $RbFe_2As_2$ are plotted against temperature in Figs. S1(a) and S1(b). In this new set of data obtained by the Montgomery method, we see that $m_{A_{1g}}$ is indeed not negligible but has only weak temperature dependence. On the other hand, if we focus on the relative temperature dependence, $m_{11} - m_{12}$ shows the strongest T dependence, which is essentially consistent with those obtained by the longitudinal elastoresistance method [6]. The increasing trend of $m_{11} - m_{12}$ with decreasing temperature implies that $RbFe_2As_2$ exhibits the nematic instability for Fe-

As diagonal directions. Moreover, we observe a kink-like feature at $T_{\rm nem} \simeq 47$ K, signaling the nematic transition. These results are consistent with our thermodynamic evidence for the two-fold symmetry at low temperatures as well as the results of Mössbauer spectroscopy.

III. ALIGNMENT OF THE MAGNETIC FIELD

The misalignment of the magnetic field against the ab-plane of the sample is adjusted by controlling independently the currents of two superconducting coils implemented in a vector magnet. Figure S2 shows the C/T magnitude as a function of θ_0 and ϕ, where θ_0 is the initial polar angle determined by the vertical magnet axis. The maximum positions of C/T along θ_0 follow the sinusoidal dependence on ϕ with the phase irrelevant to the crystallographic axes, which is the typical angular dependence expected when the c axis of the sample has some misalignment from the magnet axis. The magnitude of the misalignment is typically 2-3°, which is estimated from the amplitude of the sinusoidal curve. The misalignment can be adjusted to less than 0.5° after the calibration procedure, as shown in the Fig. 2(c) of the main text.

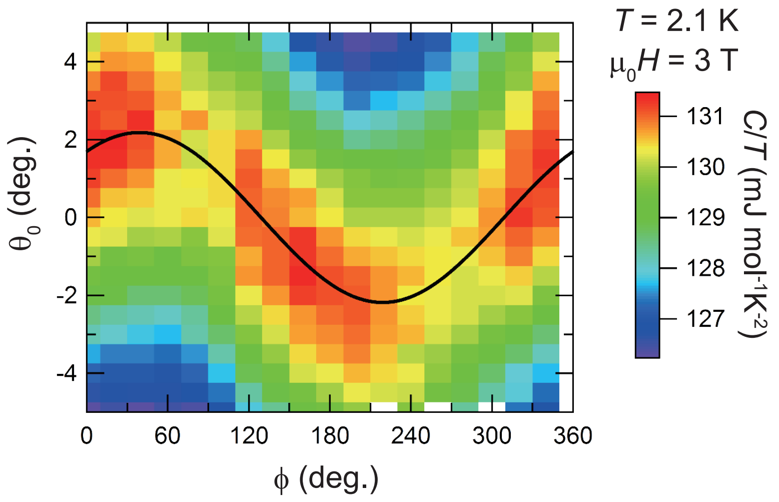

FIG. S2. Raw data of angular dependence of C/T without alignment of the magnetic field at 3 T and 2.1 K. Black solid line represents the ϕ dependence of the maximum position of $C/T(\theta_0)$.

IV. FITTING OF $C/T(T,\phi)$ FOR 2 T

The fitting of the $C/T(T,\phi)$ data for 2 T is carried out using Eq.(1) in the main text. To model the heat capacity jump at T_c with finite broadness, we employ the following function for each domain $i = 1, 2$;

$$C_i/T(T;T_{ci}) = \frac{A_sT + B_s}{2}\left(1 + \arctan\frac{T_{ci} - T}{\delta}\right) + \frac{A_nT + B_n}{2}\left(1 - \arctan\frac{T_{ci} - T}{\delta}\right). \quad \text{(S5)}$$

Here, the T-dependence of C/T above T_c is approximated by the term $A_nT + B_n$ with parameters A_n and B_n, and that below T_c by $A_sT + B_s$ with parameters A_s and B_s. The parameter δ determines the broadness of the superconducting transition. The mid-point transition temperature T_{ci} has the two-fold angular dependence as discussed in the main text. Figure S3 depicts the color map of the calculated $C/T(T,\phi)$ with parameters that fit the data. The characteristic two-fold term in amplitude is in excellent agreement with the experimental data shown in Fig. 3(b) of the main text, indicating that our model captures the essential features of the observed angular dependence of the specific heat.

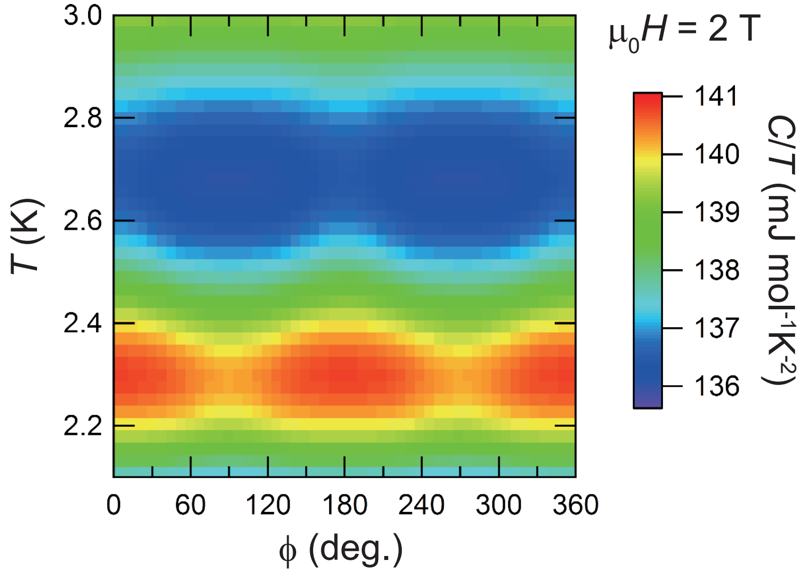

FIG. S3. Calculated $C/T(T,\phi)$ from the fitting for the specific heat data for 2 T.

V. ANALYSIS OF MÖSSBAUER QUADRUPOLE SPLITTING

The T-dependence of the quadrupole splitting 2ε at high temperatures is mainly determined by the energy gap Δ between d_{xy} and $d_{xz}(d_{yz})$ orbitals in the tetragonal phase of iron pnictides with space group $I4/mmm$. In the nematic state, the in-plane anisotropy can give additional temperature dependence in 2ε associated with the split of the gap Δ. When the gap splitting is small compared with Δ, the quadrupole splitting 2ε and the asymmetric (nematic order) parameter $\eta(T)$ can be related through $2\varepsilon \propto F(T)(1+\eta^2(T)/3)^{1/2}$ [8]. Here, the $F(T)$ is T-dependent prefactor and gives the temperature dependence of 2ε above the nematic transition with $\eta(T) = 0$. Then, we assume that $(2\varepsilon)^2$ can be described as $(2\varepsilon)^2 = A + BF^2(T)(1+\eta^2(T)/3)$ where A gives the T-independent contribution, B is the magnitude of the T-dependent term. To subtract the component irrelevant to the asymmetric parameter, we fit the $(2\varepsilon)^2$ at high temperatures to the function based on Ref. [8]

$$\begin{aligned}(2\varepsilon(T))^2 &= A + BF^2(T)\\ &= A + B\left(\left(1-\mathrm{e}^{-\frac{\Delta}{k_BT}}\right)/\left(1+2\mathrm{e}^{-\frac{\Delta}{k_BT}}\right)\right)^2,\end{aligned} \tag{S6}$$

where k_B is the Boltzmann constant. The fitting result using Eq. (S6) is shown in Fig. S4, and we obtain the energy gap $\Delta/k_B = 206$ K, which is close to the typical value in the tetragonal

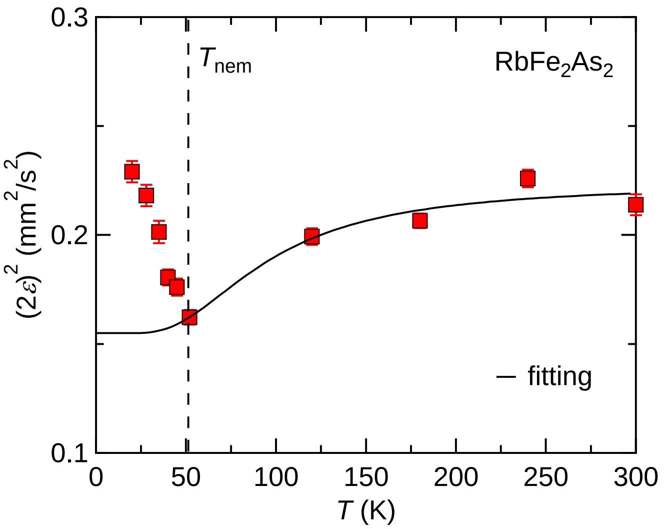

FIG. S4. Analysis of the T-dependence of quadrupole splitting at high temperatures. Black solid line represents the fitting curve given by Eq. (S6). The dashed line indicates the onset of the nematic order T_{nem}.

phase of iron pnictides within the ambiguity due to the momentum dependence [9]. By subtracting this high-temperature component $(2\varepsilon_{\rm high})^2$, we obtain $[(2\varepsilon)^2$ - $(2\varepsilon_{\rm high})^2]^{1/2} \propto F(T)\eta(T)$, whose temperature dependence is approximated by $\eta(T)$ below $T_{\rm nem}$, where the T-dependence in $F(T)$ is negligibly small as shown in Fig. S4.

---

[1] K. Kihou, T. Saito, S. Ishida, M. Nakajima, Y. Tomioka *et al.*, Single Crystal Growth and Characterization of the Iron-Based Superconductor KFe_2As_2 Synthesized by KAs Flux Method, J. Phys. Soc. Jpn. **79**, 124713 (2010).

[2] Y. Mizukami, M. Haze, O. Tanaka, K. Matsuura, D. Sano *et al.*, Unusual crossover from Bardeen-Cooper-Schrieffer to Bose-Einstein-condensate superconductivity in iron chalcogenides, Communications Physics **6**, 183 (2023).

[3] T. Mitsui, N. Hirao, Y. Ohishi, R. Masuda, Y. Nakamura *et al.*, Development of an energy-domain ^{57}Fe-Mössbauer spectrometer using synchrotron radiation and its application to ultrahigh-pressure studies with a diamond anvil cell, J. Synchrotron Radiat. **16**, 723-729 (2009).

[4] P. Wiecki, A.-A. Haghighirad, F. Weber, M. Merz, R. Heid *et al.*, Dominant In-Plane Symmetric Elastoresistance in $CsFe_2As_2$, Phys. Rev. Lett. **125**, 187001 (2020).

[5] P. Wiecki, M. Frachet, A.-A. Haghighirad, T. Wolf, C. Meingast *et al.*, Emerging symmetric strain response and weakening nematic fluctuations in strongly hole-doped iron-based superconductors, Nat. Commun. **12**, 4824 (2021).

[6] K. Ishida, M. Tsujii, S. Hosoi, Y. Mizukami, S. Ishida *et al.*, Novel electronic nematicity in heavily hole-doped iron pnictide superconductors, Proc. Natl. Acad. Sci. U. S. A. **117**, 6424-6429 (2020).

[7] J. Park, J. M. Bartlett, H. M. L. Noad, A. L. Stern, M. E. Barber *et al.*, Rigid platform for applying large tunable strains to mechanically delicate samples, Rev. Sci. Instrum. **91**, 083902 (2020)

[8] Y. Li, J. Xue, S. Hu, and H. Pang, Mössbauer spectroscopy study of nematicity in $Ba(Fe_{0.962}Cu_{0038})_2As_2$ single crystal: enhanced orbital effect, J. Phys.: Condens. Matter **33**, 205602 (2021).

[9] M. F. Jensen, V. Brouet, E. Papalazarou, A. Nicolaou, A. Taleb-Ibrahimi *et al.*, Angle-resolved photoemission study of the role of nesting and orbital orderings in the antiferromagnetic phase

of $BaFe_2As_2$, Phys. Rev. B **84**, 014509 (2011).
